# Individualized Nutritional Counseling Closes Growth and Nutrition Gaps in Infants With Food Protein‐Induced Allergic Proctocolitis: 6‐Month Follow‐Up Study

**DOI:** 10.1002/fsn3.70498

**Published:** 2025-06-20

**Authors:** Menşure Nur Çelik, Eda Köksal

**Affiliations:** ^1^ Ondokuz Mayis University Department of Nutrition and Dietetics Samsun Turkey; ^2^ Gazi University Department of Nutrition and Dietetics Ankara Turkey

**Keywords:** calcium, cow's milk protein allergy, elimination diet, food consumption, FPIAP, milk and dairy products, nutrition

## Abstract

The aim of this study was to evaluate the growth and nutritional intake of infants with food protein‐induced allergic proctocolitis (FPIAP) on a therapeutic elimination diet until 1 year of age, compared to healthy controls. This case–control study followed 13 FPIAP infants and 22 healthy controls from 6 to 12 months. FPIAP infants and their mothers adhered to a cow's milk protein elimination diet. All mothers received complementary feeding education; FPIAP mothers had individualized dietitian counseling. Growth and nutritional intake were assessed at 6, 7, 9, and 12 months. During the study, the weight‐for‐age of infants with FPIAP was found to be lower compared to healthy controls (*p* < 0.05). Height‐for‐age was shorter in infants with FPIAP than in controls at the beginning of the study (*p* < 0.05), and this difference disappeared when the infants were 12 months old (*p* > 0.05). During and after the elimination diet, total protein (g) and animal protein intakes of infants with FPIAP were lower than those of controls (*p* < 0.05). Similarly, calcium, phosphorus, and vitamin B2 intakes were lower in the FPIAP group during and after the elimination diet (*p* < 0.05). Individualized nutritional counseling supports adequate growth and nutrition in FPIAP infants, highlighting the importance of breast milk continuity and tailored complementary feeding.

## Graphical abstract

Infants with FPIAP on a cow's milk protein elimination diet often experience growth delays and inadequate intake of protein, calcium, and vitamin B2, which can affect development without nutritional support. Individualized nutritional counseling improves growth and nutrient intake in breastfed FPIAP infants, closing growth differences with healthy peers by 12 months. FPIAP, Food Protein‐İnduced Allergic Proctocolitis.

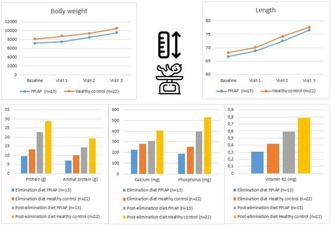

## 1 Introduction

Food protein‐induced allergic proctocolitis (FPIAP) is a cell‐mediated inflammation of the distal sigmoid colon and rectum that usually occurs in the first months of infancy. Exclusively breastfed infants account for more than 50% of all FPIAP cases, and cow's milk protein (CMP) plays a notable role in this process (Labrosse et al. 2020; Mennini et al. 2020; Meyer et al. 2020; Nowak‐Węgrzyn 2015). Although the exact prevalence is unknown, the most common type of non‐IgE‐mediated cow's milk protein allergy (CMPA) is FPIAP (Elizur et al. 2012). The prevalence of CMPA in the first year of life is estimated to be 2%–7.5% (Agostoni et al. 2009; Boyce et al. 2011; Høst 2002). Turkey has no nationwide epidemiological studies (Guler et al. 2020).

After confirming a cow's milk protein allergy (CMPA) diagnosis, an elimination diet should be maintained for at least 6 months or until the infant reaches 9–12 months. Although CMPA typically resolves by ages 1–2, evidence remains insufficient to establish the best timing for retesting (Koletzko et al. 2012).

Cow's milk contains essential macro and micronutrients necessary for normal growth and development, especially in early childhood. Research shows that dairy products positively affect bone health during childhood and adolescence. Therefore, it is thought that eliminating cow's milk may cause some deficiencies (Rozenberg et al. 2016). Children with CMPA are at risk of developing certain micronutrient deficiencies, such as calcium and vitamin D (Foong et al. 2017; Noimark and Cox 2008). Research shows that infants with CMPA consume less energy, fat, protein, and calcium than healthy controls (Sova et al. 2013; Tuokkola et al. 2010). It was hypothesized that individualized nutritional counseling and appropriate dietary interventions could mitigate growth and nutritional deficiencies in FPIAP infants and close the gap with healthy controls by 12 months of age. This research aimed to evaluate the growth and nutritional intake of 6‐month‐old FPIAP infants receiving a therapeutic elimination diet until the first year of life and to compare them with healthy controls. We hypothesized that individualized nutritional counseling can mitigate growth and nutritional deficiencies in FPIAP infants and close the gap with healthy controls by 12 months.

## 2 Method

### 2.1 Study Design and Population

In this prospective case–control study, 6‐month‐old infants diagnosed with FPIAP (diagnosed by a specialist physician) who were breastfed and applied a cow's milk elimination diet and healthy term infants in the same age range who were breastfed and fed appropriately for their age and who did not apply any elimination diet were evaluated. Power analysis was carried out using the Zeng et al. (2019) (Zeng et al. 2019) study as a guide. Using a 95% power (power = 0.95) and 5% significance level (*α* = 0.05) for the computation, it was concluded that 11 infants per group would be statistically sufficient.

In this study, which was conducted as a prospective case–control study, 6‐month‐old FPIAP diagnosed infants who were breastfeed and applied a cow's milk elimination diet and healthy term infants who were breastfed fed age‐appropriatelyand who were not followed any elimination diet in the same age range were evaluated. The data for the study was collected between August 2020 and March 2022 by meeting face‐to‐face with the mothers of the infants in the sample group. The general plan of the study is given in Supporting Information S1.

### 2.2 Eligibility Criteria

**FIGURE 1:**
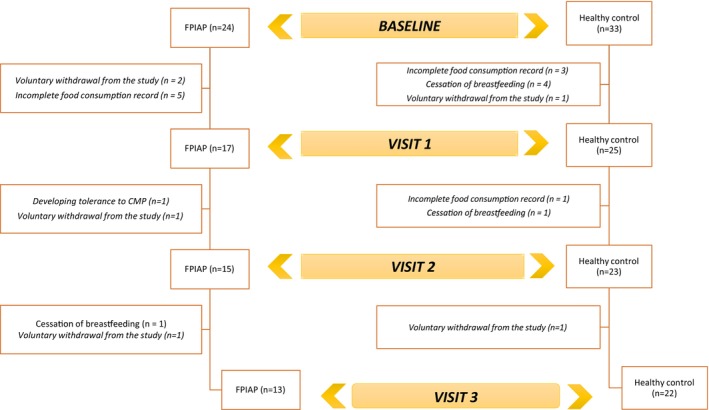
Flowchart of participant inclusion and withdrawal.

Infants aged 17–26 weeks who were breastfed between the study dates and diagnosed with CMPAP by a physician, infants with a gestational age of 37–42 weeks and singleton birth, infants with a birth weight ≥ 2500 and ≤ 4500 g, and healthy term infants aged 17–26 weeks who were breastfed were included in the study. The control group consisted of healthy term infants aged 17–26 weeks who were exclusively breastfed, had no history of food allergy or gastrointestinal symptoms, and met the same criteria for gestational age, birth weight, and singleton birth. Premature births, multiple pregnancies, those with a known chronic or systemic disease diagnosed, those with neonatal disease or congenital malformations, those who had an infection or disease requiring hospitalization during the study period, those who never received breast milk, and mothers who did not want to continue after participating in the study and their infants were excluded. The process of including infants in the study and their withdrawal from the study for various reasons is schematized in Figure 1.

### 2.3 Anthropometric Measurements and Evaluation

Body weight and length of all infants were measured by the same dietitian and with the same measuring instruments throughout the study period. The body weight of the infants was measured with a standard baby scale (Seca, Germany) sensitive to 10 g while the infants were completely naked. The length was measured with an infantometer (Seca, Germany), which has a tape measure on one side and a movable section that allows the infants' feet to be fixed in the supine position.

The study began when 6‐month‐old infamnts were on an elimination diet (Baseline). Body weight and length were measured by the technique at the beginning of the study, after 4 weeks (Visit 1), when the elimination diet ended (when the infants were 9 months old) (Visit 2), and when the infants were 12 months old (Visit 3) (Centers for Disease Control and Prevention 2007). WHO Anthro program was used to calculate the *z* score of infants' weight for age *z* score (WAZ), length for age *z* score (LAZ) and weight for length *z* score (WLZ) (World Health Organization 2006).

### 2.4 The Elimination Diet Procedure

After applying to Gazi University Health Application and Research Center, Department of Pediatric Allergy and Asthma and receiving a CMPA diagnosis from a specialist physician, the principles of cow's milk protein elimination were explained to the mothers in detail (Table 1). Complementary feeding practices are further detailed in the Supporting Information, including a sample monthly feeding program (S2) and feeding guidelines on frequency, quantity, and texture (S3). During the study, diet planning for infants was done under the guidance of a dietician, using Dietary Reference Intakes (DRI) as areference.

**TABLE 1:** Nutrients and components to be eliminated in cow's milk protein elimination.

Food	Ingredients
Milk (cow, goat, sheep, and other animal milk) Yoghurt Kefir Margarine Butter Cheese (mild cream cheese, strained, curd, kashar cheese, tulum) Cream Pudding Cream Kastırd Ice cream Biscuits Wafers Crackers Bechamel sauce Milk desserts (muhallebi, rice pudding, keskül, kazandibi etc.) Cake, muffin Coffee cream Milk powder Whipped cream Boza	Milk protein hydrolysate Casein Casein hydrolysate Caseinate Rennet casein Whey Whey protein hydrolysate Lactulose Diacetyl Lactalbumin Lactalbumin phosphate Lactitol monohydrate Lactoferrin Tagatose (a type of sweetener) Whey Lactose Galactose Lactulose Diacetyl Rekaldent

In the study, an elimination diet was applied to infants diagnosed with FPIAP and their mothers under the supervision of a dietitian until the infants were 9 months old. From the 9th month onwards, the milk ladder started the diet (Luyt et al. 2014). First of all, the mother's diet began to be opened, and if it did not cause any symptoms in the infant, the infant's diet was also opened by the milk ladder. When infants reach the age of 1, dietary elimination is wholly terminated. The periods when infants with FPIAP were on the elimination diet (baseline and Visit 1) were classified as “elimination diet,” and the periods when the elimination diet was ended (Visit 2 and Visit 3) were classified as “post‐elimination diet.”

### 2.5 Maternal Elimination Diet

After mothers were given general information about the characteristics of the elimination diet, an appropriate nutrition program was planned, and information was given about the foods and components they needed to eliminate. The importance of breast milk was explained, and mothers were encouraged to breastfeed. In addition, a list of the diet's prohibitions was given along with the planned nutrition program (Table 1) (Altintaş et al. 2017). In addition, calcium (1000 mg/day) and vitamin D (1000 IU/day) supplements were administered to all mothers by the physician.

### 2.6 Determining the Food Consumption of Infants

To evaluate the nutritional status of the infants, mothers were asked to keep a 3‐day food consumption record for their infants (2 days on weekdays and 1 day on the weekend) at each check‐up. Detailed training was given to mothers on how to keep a record of food consumption. The types and amounts of daily consumed foods were determined, and energy, macro, and micronutrient values were calculated using the BeBİS version 9.0 program prepared for Turkish foods. BeBİS 9.0 is a software that is widely used in the analysis of energy and nutrient content of meals in Türkiye and is frequently used in nutrition‐related research. It combines databases of German Nutrient, the US Department of Agriculture, and Turkish Food Composition, contributions of all ingredients.

### 2.7 Calculation of Breast Milk Intake of Infants

In the 3‐day food consumption records requested from mothers at each visit, they were asked to write down the amount of breast milk if they expressed it and the duration of each breastfeeding if they breastfed it. The amount of milk produced by breastfeeding mothers was evaluated using a method recommended by the cworking group. Accordingly, if the breastfeeding period lasts 10 min or longer than 10 min, the amount of breast milk is 100 mL; If the breastfeeding duration is less than 10 min, it is accepted as 10 mL/min (Emmett et al. 2000; Golding, Pembrey, and Team 2001).

### 2.8 Statistical Analyses

The data obtained from the study were analyzed using IBM SPSS version 22.0. Normality was assessed through the Kolmogorov–Smirnov test, histogram, coefficient of variation, skewness, and kurtosis. For comparisons between two independent groups, the Independent Sample *t*‐test was applied when normal distribution was met, whereas the Mann–Whitney *U* test was used for non‐normal distributions. The Wilcoxon Signed Rank test was employed to assess changes over time in non‐normally distributed independent groups. All statistical analyses were conducted at a significance level of *p* < 0.05 with a 95% confidence interval.

## 3 Results

In this study, the average duration of stay on the elimination diet of 13 FPIAP infants was determined to be 5.8 ± 1.0 months, and the time to end the elimination diet was 8.6 ± 1.0 months. It was found that 69.2% of infants with FPIAP started complementary feeding on time, and 30.8% started late (not shown in the table).

A comparison of infants' anthropometric measurements and indices is given in Table 2. During the study, the mean body weight of infants with FPIAP was lower than that of healthy controls (*p* < 0.05). The mean length of infants was shorter in FPIAP than that of healthy controls throughout the study period, and this difference was found to be significant only at baseline and Visit 2 (*p* < 0.05). There was a statistically significant difference between infants with FPIAP and healthy controls at baseline, Visit 1, and Visit 3 only in terms of WAZ and WLZ values (*p* < 0.05 for each) (Table 2).

**TABLE 2:** Comparison of infants' anthropometric measurements and indexes during follow‐up.

	FPIAP (*n* = 13)	Healthy control (*n* = 22)	*p*
	$\bar{x}$ ± SD	$\bar{x}$ ± SD	
BW (g)			
Birth weight	3405.7 ± 324.8	3366.5 ± 334.4	0.737
Baseline	7115.8 ± 636.1	8097.7 ± 978.3	0.003*
Visit 1	7492.3 ± 658.4	8735.5 ± 1289.1	0.001*
Visit 2	8529.2 ± 707.1	9409.1 ± 1177.9	0.020*
Visit 3	9562.3 ± 739.5	10556.8 ± 1211.9	0.005*
BW increase (0–6 months)	3710.0 ± 768.8	4715.2 ± 941.6	0.003*
BW increase (6–7 months)	376.5 ± 129.3	665.0 ± 459.0	0.005*
BW increase (6–9 months)	1413.4 ± 670.4	1311.36 ± 507.7	0.613
BW increase (7–9 months)	1036.9 ± 618.2	710.0 ± 390.7	0.287
BW increase (9–12 months)	1033.1 ± 393.2	1147.7 ± 670.3	0.880
BW increase (6–12 months)	2446.5 ± 747.5	2522.7 ± 854.6	0.792
Length (cm)			
Birth length	50.38 ± 1.4	50.82 ± 1.6	0.445
Baseline	66.7 ± 0.9	68.2 ± 2.2	0.015*
Visit 1	68.8 ± 1.8	70.1 ± 2.4	0.099
Visit 2	72.5 ± 1.9	74.2 ± 2.5	0.044*
Visit 3	76.6 ± 2.1	77.7 ± 2.5	0.216
Length increase (0–6 months)	16.3 ± 1.6	17.3 ± 2.5	0.161
Length increase (6–7 months)	2.2 ± 1.2	2.0 ± 0.8	0.562
Length increase (6–9 months)	5.8 ± 1.7	6.0 ± 1.8	0.754
Length increase (7–9 months)	3.7 ± 2.1	4.0 ± 1.6	0.287
Length increase (9–12 months)	4.1 ± 1.4	3.5 ± 1.6	0.246
Length increase (6–12 months)	9.9 ± 2.0	9.5 ± 1.7	0.515
WAZ			
Birth	0.32 ± 0.80	0.10 ± 0.66	0.397
Baseline	−0.48 ± 0.75	0.38 ± 1.01	0.013*
Visit 1	−0.42 ± 0.73	0.63 ± 1.25	0.009*
Visit 2	0.07 ± 0.68	0.67 ± 1.09	0.081
Visit 3	0.33 ± 0.67	0.97 ± 0.98	0.049*
LAZ			
Birth	0.94 ± 0.88	0.87 ± 0.48	0.092
Baseline	0.15 ± 0.56	0.54 ± 1.04	0.218
Visit 1	0.42 ± 0.88	0.73 ± 1.12	0.403
Visit 2	0.75 ± 0.80	1.23 ± 1.08	0.176
Visit 3	0.82 ± 0.85	1.03 ± 1.05	0.538
WLZ			
Birth	−0.58 ± 1.13	−0.24 ± 0.78	0.352
Baseline	−0.69 ± 0.99	0.19 ± 1.14	0.028*
Visit 1	−0.78 ± 1.03	0.41 ± 1.40	0.012*
Visit 2	−0.34 ± 1.02	0.18 ± 1.24	0.207
Visit 3	−0.04 ± 1.01	0.70 ± 1.03	0.046*

Abbreviations: BW, body weight; FPIAP, food protein‐induced allergic proctocolitis; LAZ, length for age *z* score; WAZ, weight for age *z* score; WLZ, weight for length *z* score.

* *p* < 0.05.

The time when infants start complementary foods is given in Table 3. The difference between FPIAP and healthy infants in terms of the time of starting yoghurt, cheese, and tarhana soup, which contain cow's milk protein, was found to be statistically significant (*p* < 0.05). In addition, infants with FPIAP started eating red meat and fish later than those in the healthy group (*p* < 0.05). There was no significant difference between the groups regarding the start time of other foods (*p* > 0.05).

**TABLE 3:** Information on when infants start complementary foods (months).

	*n* ^a^	FPIAP (*n* = 13)	Healthy control (*n* = 22)	*p*
		$\bar{x}$ ± SD	$\bar{x}$ ± SD	
Fruit juice	FPIAP (*n* = 9) Healthy control (*n* = 16)	7.56 ± 2.01	7.06 ± 1.69	0.718
Fruit puree	FPIAP (*n* = 13) Healthy control (*n* = 22)	6.0 ± 0.00	6.05 ± 0.38	0.827
Pudding^b^	FPIAP (*n* = 11) Healthy control (*n* = 16)	7.73 ± 2.28	7.75 ± 2.21	0.942
Yoghurt	FPIAP (*n* = 11) Healthy control (*n* = 22)	9.45 ± 2.16	6.0 ± 0.44	0.000*
Cheese	FPIAP (*n* = 11) Healthy control (*n* = 22)	10.36 ± 1.80	6.86 ± 0.99	0.000*
Kefir	FPIAP (*n* = 2) Healthy control (*n* = 13)	10.50 ± 2.12	8.62 ± 2.40	0.381
Tarhana soup	FPIAP (*n* = 10) Healthy control (*n* = 21)	8.90 ± 2.33	7.10 ± 1.48	0.000*
Vegetable soup/broth	FPIAP (*n* = 13) Healthy control (*n* = 22)	6.23 ± 0.44	6.86 ± 1.46	0.159
Vegetable puree	FPIAP (*n* = 13) Healthy control (*n* = 22)	6.31 ± 0.48	6.18 ± 0.59	0.489
Egg yolk	FPIAP (*n* = 10) Healthy control (*n* = 22)	7.00 ± 1.21	6.50 ± 1.14	0.179
Whole egg	FPIAP (*n* = 11) Healthy control (*n* = 22)	9.82 ± 2.32	9.48 ± 2.02	0.668
Inside of bread	FPIAP (*n* = 13) Healthy control (*n* = 22)	8.31 ± 2.32	7.91 ± 1.60	0.591
Rice	FPIAP (*n* = 13) Healthy control (*n* = 22)	7.23 ± 1.24	8.23 ± 2.07	0.125
Red meat/minced meat	FPIAP (*n* = 13) Healthy control (*n* = 22)	8.54 ± 1.51	7.36 ± 1.14	0.013*
Chicken meat	FPIAP (*n* = 13) Healthy control (*n* = 22)	9.0 ± 1.18	8.06 ± 1.51	0.089
Fish	FPIAP (*n* = 13) Healthy control (*n* = 22)	10.45 ± 1.29	8.20 ± 1.44	0.000*
Lentil soup	FPIAP (*n* = 13) Healthy control (*n* = 22)	7.85 ± 1.34	8.14 ± 1.36	0.544
Dry beans	FPIAP (*n* = 11) Healthy control (*n* = 21)	8.64 ± 1.36	8.67 ± 1.65	0.959

^a^ Number of infants to whom foods were introduced during the study.

^b^ In the FPIAP group, milk and dairy products were not used in pudding.

* *p* < 0.05.

Accordingly, total protein (g), animal protein (g), riboflavin, calcium, and phosphorus intakes during and after the elimination diet were found to be significantly lower in the FPIAP group compared to healthy controls (*p* < 0.05). The proportion of energy coming from carbohydrates was found to be higher in the FPIAP group than in healthy controls, both during the elimination diet (*p* < 0.05) and after the elimination diet (*p* > 0.05). There was no difference between the groups during and after the elimination diet regarding the ratio of energy from protein and fat (*p* > 0.05) (Table 4).

**TABLE 4:** Comparison of daily energy and macronutrient intake levels of infants during and after the elimination diet^a^.

	Elimination diet			Post‐elimination diet		
	FPIAP (*n* = 13)	Healthy control (*n* = 22)	*p*	FPIAP (*n* = 13)	Healthy control (*n* = 22)	*p*
Total energy (kcal)	573.36 ± 119.53	653.52 ± 142.86	0.085	781.26 ± 231.60	901.86 ± 180.94	0.095
Total energy (kcal/kg)	79.70 ± 21.49	78.18 ± 16.91	0.818	87.23 ± 29.77	90.76 ± 20.10	0.677
Carbohydrate (g)	62.69 ± 13.40	66.87 ± 12.67	0.362	85.10 ± 30.46	88.81 ± 20.58	0.053
Carbohydrates (%)	50.74 ± 15.52	41.97 ± 2.68	0.024*	45.58 ± 8.09	40.40 ± 4.87	0.053
Protein (g)	9.48 ± 3.15	13.21 ± 4.73	0.003*	22.75 ± 6.83	28.70 ± 7.89	0.031*
Protein (g/kg)	1.31 ± 0.51	1.58 ± 0.60	0.169	2.53 ± 0.89	2.89 ± 0.93	0.273
Protein (%)	7.97 ± 3.72	7.97 ± 1.65	0.139	12.30 ± 2.33	12.95 ± 2.33	0.489
Vegetable protein (g)	2.50 ± 1.90	3.07 ± 2.33	0.601	8.42 ± 2.62	9.51 ± 2.83	0.268
Animal protein (g)	6.98 ± 2.11	10.13 ± 3.00	0.001*	14.33 ± 5.05	19.18 ± 6.93	0.035*
Fat (g)	31.97 ± 7.09	37.04 ± 8.87	0.089	38.37 ± 12.02	47.28 ± 10.50	0.028*
Fat (%)	56.83 ± 19.93	50.09 ± 2.65	0.880	46.21 ± 9.90	46.52 ± 3.52	0.775
Vitamin A (mcg)	578.75 ± 188.02	614.59 ± 221.95	0.987	726.13 ± 255.86	736.91 ± 266.52	0.907
Vitamin B1 (mg)	0.33 ± 0.46	0.24 ± 0.09	0.649	0.42 ± 0.22	0.44 ± 0.12	0.139
Vitamin B2 (mg)	0.31 ± 0.09	0.42 ± 0.13	0.005*	0.59 ± 0.21	0.79 ± 0.21	0.013*
Niacin (mg)	1.59 ± 1.05	2.64 ± 1.57	0.034*	7.21 ± 2.53	8.65 ± 3.23	0.178
Vitamin B6 (mg)	0.47 ± 0.47	0.41 ± 0.12	0.335	0.69 ± 0.29	0.76 ± 0.23	0.418
Vitamin B12 (mcg)	151.84 ± 45.80	217.09 ± 70.33	0.002*	370.53 ± 119.07	423.72 ± 145.54	0.273
Calcium (mg)	224.92 ± 76.31	282.17 ± 82.01	0.006*	309.88 ± 130.61	404.44 ± 119.35	0.036*
Phosphorus (mg)	186.91 ± 70.75	253.18 ± 102.31	0.016*	398.20 ± 127.78	528.43 ± 146.29	0.012*
Iron (mg)	1.70 ± 1.24	1.84 ± 1.03	0.578	4.49 ± 1.96	4.51 ± 1.60	0.699
Zinc (mg)	1.90 ± 0.64	2.31 ± 0.83	0.191	3.71 ± 0.99	4.44 ± 1.22	0.076

^a^ The periods when infants with FPIAP are on the elimination diet (initial and 1st visit) refer to the “elimination diet,” and the periods when the elimination diet is started (2nd and 3rd visit) refer to the “post‐elimination diet.”

* *p* < 0.05.

A comparison of the consumption amounts (g) of infants from food groups during and after the elimination diet is given in Table 5. Consumption of milk and its products (g) was found to be lower in infants with FPIAP during and after the elimination diet (*p* < 0.05). When the difference between the consumption amounts of food groups during and after the elimination diet was evaluated within the group, a statistically significant increase was determined after the elimination diet in all food groups in both FPIAP infants and healthy infants (*p* < 0.05).

**TABLE 5:** Comparison of food group consumption amounts (g) of infants during and after the elimination diet.

Food groups (g)	Elimination diet			Post‐elimination diet			*p* ^FPIAP^	*p* ^control^
	FPIAP (*n* = 13)	Healthy control (*n* = 22)	*p* ^α^	FPIAP (*n* = 13)	Healthy control (*n* = 22)	*p* ^β^		
Breast milk (mL)	573.27 ± 61.49	634.17 ± 124.26	0.109	382.05 ± 119.64	420.55 ± 151.83	0.441	0.000*	0.000*
Milk and dairy products (g)	5.34 ± 18.97	49.97 ± 45.15	0.000*	58.46 ± 66.53	127.43 ± 78.66	0.004*	0.003*	0.000*
Egg (g)	4.26 ± 4.31	7.79 ± 5.16	0.018*	25.80 ± 14.56	32.00 ± 13.67	0.215	0.002*	0.000*
Meat, chicken, fish (g)	1.53 ± 3.15	4.15 ± 5.38	0.106	26.57 ± 20.17	29.15 ± 23.98	0.933	0.001*	0.000*
Legumes (g)	0.11 ± 0.41	2.18 ± 5.22	0.319	4.92 ± 4.57	5.22 ± 4.76	0.933	0.006*	0.022*
Oilseeds (g)	0.96 ± 2.01	0.79 ± 2.82	0.555	1.26 ± 1.77	2.09 ± 3.15	0.749	0.233	0.028*
Bread and cereals (g)	9.19 ± 11.44	8.93 ± 8.22	1.000	38.26 ± 15.41	41.63 ± 18.77	0.880	0.003*	0.000*
Fresh vegetables (g)	41.80 ± 26.02	44.47 ± 32.69	0.724	91.42 ± 49.90	93.20 ± 50.01	0.919	0.003*	0.000*
Fresh fruit (g)	53.19 ± 35.78	50.70 ± 31.58	0.832	102.76 ± 67.00	113.15 ± 63.04	0.699	0.005*	0.000*
Fat (g)	2.15 ± 2.06	4.18 ± 3.73	0.049*	11.15 ± 4.69	14.77 ± 7.33	0.121	0.002*	0.000*
Sugar (molasses) (g)	1.84 ± 2.56	1.72 ± 2.38	0.880	4.34 ± 4.70	4.25 ± 4.97	0.749	0.124	0.011*

Abbreviations: *p* ^control^, Change in the control group during and after the elimination diet; *p* ^FPIAP^, Change in the FPIAP group during and after the elimination diet; *p* ^α^, Comparison of two groups during the elimination diet; *p* ^β^, Comparison of two groups after the elimination diet.

* *p* < 0.05.

## 4 Discussion

This study is thought to be important because it is the first longitudinally planned case–control study in our country in which the growth of FPIAP infants who received nutritional counseling, their food consumption during the complementary feeding period was compared with their healthy peers, and was followed up to the age of one. Our hypothesis that individualized nutritional counseling can mitigate growth and nutritional deficiencies in FPIAP infants and close the gap with healthy controls by 12 months was confirmed by the study findings.

This is especially important in young children with CMPA, as growth is rapid, especially in the first 2 years of life, when the prevalence of allergy is high (Barclay and Weaver 2006). It is essential to monitor the malnourished child with CMPA (Meyer et al. 2012). In one study, WAZ and WLZ of infants with CMPA following a therapeutic elimination diet from the first few months of life were significantly lower than healthy controls. However, at the end of the follow‐up period, growth catch‐up was reportedly achieved as WAZ and WLZ were similar between the two groups (Dong et al. 2018). In this study, it was observed that when the infants turned 1 year old, infants with FPIAP caught up with their healthy peers, and although the WLZ were lower than the healthy controls throughout the study, there was no difference between the groups (except Visit 2, *p* < 0.05). As length increase is important for the evaluation of long‐term nutritional status in this period when growth is rapid, close follow‐up of children with FPIAP with nutritional counseling and the effectiveness of the elimination diet may have been effective in this result. In addition, the lower body weight values observed in infants on the elimination diet were similar to those reported in previous studies (Medeiros et al. 2004; R Meyer et al. 2019). Failure to improve body weight, such as in length increase, may be due to acute reasons such as changes in physical activity that occur in the 6–12 months of life or decreased food intake during teething periods. It is important to monitor weight change rather than body weight to be able to talk about a condition caused by FPIAP. In this study, although the weight gain of infants with FPIAP was lower than that of healthy infants until the first visit, it was observed that they caught up with their healthy peers in the following periods. At this point, it is possible to talk about the positive effect of the elimination diet given with regular nutritional counseling on weight gain in infants with FPIAP.

According to national and international guidelines, complementary feeding for infants with CMPA should be the same as recommended for healthy children (Agostoni et al. 2008; Koletzko et al. 2012). One study determined the median age of introducing complementary foods as five months in the CMPA group and four months in the control group (*p* < 0.05). In the same study, a delay in the introduction of potentially allergenic foods such as eggs and wheat was observed in the CMPA group (*p* < 0.05) (Frizzo et al. 2022). In our study, all infants in the healthy control group started complementary feeding on time. Although most of the infants in the FPIAP group started on time, similar to the literature, it was also determined that some had late onset. There was no difference between the groups in terms of the time they started eating foods with a lower risk of developing allergies. This result may be due to the positive effect of complementary nutrition education given in regular meetings with the mothers of infants in the patient group and explaining to the mothers that the allergenic risk of these foods is lower.

In one study, healthy children started eating yoghurt at 8.3 months, fish at 11.3 months, and red and chicken meat at 10.7 months; legumes were found to last 12 months, and egg whites at 9.6 months. It has been determined that foods such as yoghurt and egg yolk are started late (Uzun et al. 2020). In another study, infants consumed milk and dairy products, meat, fish, chicken, eggs, fruit purees/juices, and cereals between the 10th and 11th months (Çatak et al. 2012). In this study, similar to the healthy literature, the time for infants with FPIAP to start milk and milk products was later than healthy peers, as expected. In general, red meat and fish initiation times in infants diagnosed with FPIAP are later than healthy infants, whereas chicken initiation times are similar. Mothers are thought to avoid red meat (information that it may cause allergies in these children due to cross‐reaction with cow's milk) and prefer chicken meat due to the information they obtained from social media. However, there was no significant difference between the groups in terms of the time during which the egg, which is risky for allergy, was included. Here, the fact that mothers gave eggs to their infants on time because they are an important source of protein was followed up regularly, and the nutrition education given may have been effective.

It is difficult to compare studies on dietary intake due to the use of reference ranges by different authorities in macro and micronutrient evaluations. In addition, the software in which analyses are performed varies depending on the country where the study is conducted. Therefore, the same food may have varying macro and micronutrient contents. Past studies have shown lower energy and nutrient intakes in infants fed a cow's milk elimination diet (Medeiros et al. 2004; Meyer 2018). Energy, protein, calcium, iron, phosphorus, riboflavin, niacin and zinc deficiencies have been reported in children with CMPA (Isolauri et al. 1998; Tiainen et al. 1995). In a study, the average contribution of protein to energy was 13.8%, carbohydrates 51.2%, and fats 35% (Meyer et al. 2016). Another study reported that the diets of children with allergies contained significantly more carbohydrates than healthy children's diets (Rowicka et al. 2017). Our findings in this study are similar to those of the literature in that the percentage of energy coming from carbohydrates was higher in infants with FPIAP during the elimination diet, and the percentage of energy coming from fat was higher than in the literature. During and after the elimination diet, the total protein (g) and animal protein (g) intake of FPIAP infants were significantly lower compared to healthy controls, as expected, as milk and dairy products were eliminated and gradually added to the diet, as well as meat, It is associated with lower consumption of important protein sources such as chicken and fish in the FPIAP group compared to the control group.

One study reported lower calcium and phosphorus intake in the CMP‐free diet group compared to the control group (Haack et al. 2017; Medeiros et al. 2004). There were lower B_2_, niacin, iron, and calcium intakes, and statistical differences were found between children who eliminated cow's milk protein and children who ate normally (Henriksen et al. 2000; Robbins et al. 2014). In this study, calcium and phosphorus intakes during and after the elimination diet are similar to the literature in that they are lower in infants with FPIAP than in the control group. Still, there are variable situations in terms of other micronutrients. These low intake amounts seen during the elimination diet in infants with FPIAP are expected due to the elimination of milk and dairy products, which are sources of calcium and phosphorus, as stated in the literature.

After the elimination diet, the decrease in calcium and phosphorus intake compared to healthy controls continued due to the gradual addition of milk and its products. In addition, as expected, the low intake of vitamin B_2_ during and after the elimination diet is due to insufficient intake of milk and dairy products. It is thought that the differences in other micronutrients are due to the difference in the consumption amount of breast milk and food groups. In addition, it was reported in another study that there may be a decrease in vitamin B_12_ intake and calcium and phosphorus intake compared to the control group due to the decrease in milk and dairy product intake in patients who developed eating behavior disorders after an elimination diet (Ercan and Tel Adıgüzel 2022).

In a study evaluating the consequences of food intake and nutritional status following discontinuing a cow's milk‐free diet after an oral nutritional challenge, children's energy, protein, carbohydrate, calcium, and phosphorus intake increased after discontinuing the elimination diet (de Faria et al. 2022). After the elimination diet was ended, this study showed similar increases in macro and micronutrient intakes in the patient group. These increases are due to the increased amounts consumed as the months progressed in both groups, and the termination of the elimination diet was also effective in the increases in infants with FPIAP.

No study has been found in the literature evaluating exclusive breast milk consumption in infants with CMPA. In a study evaluating infants with CMPA who were partially or exclusively breastfed and healthy controls, it was found that the daily milk consumption (including breast milk and hypoallergenic formula) of infants with CMPA at the age of 6 months was lower than that of infants in the control group (Shah et al. 2014). Accordingly, similar to the literature, in this study, the amount of breast milk intake was lower in infants with FPIAP than in healthy controls during the elimination diet.

A study comparing the intake of each food ingredient to the recommended dietary intake in allergic children found that most children with food allergies had intakes of food groups such as dairy products, meat, fruits, vegetables, and grains much lower than the recommended standard (Flammarion et al. 2011). The results in this study are similar. This result is expected due to the limitations of the elimination diet. In addition, egg consumption, an important protein source for infants in this age group, and other food groups were lower in FPIAP infants compared to healthy controls during and after the elimination diet. The difference in consumption amounts in terms of these food groups is a significant factor in the food selection of mothers of allergic infants compared to healthy infants. It is predicted that this may be due to mothers being more selective or their food intake is affected by the symptoms experienced by infants due to allergies.

The study's strengths include its importance as one of the first longitudinally designed case control studies in which the growth parameters of FPIAP infants throughout the study, given diet counseling, were examined by comparing them with their healthy peers during and after the elimination diet. The fact that food consumption records were collected covering all four seasons of the year allowed the evaluation of the effect of seasonal differences. The fact that a single researcher collected the data minimized measurement bias. The fact that 3‐day food consumption records were taken at two different time points, before and after the elimination diet, contributed to a more precise evaluation of the effect of changes in food intake on growth. This study emphasizes the importance of policies supporting the continuation of breastfeeding and the need to disseminate complementary feeding education to prevent nutritional deficiencies in early childhood. The results showed that although body weight values were lower in infants who applied the cow's milk elimination diet compared to the control group, growth could be supported thanks to appropriate complementary feeding practices, and high food intake and nutritional counseling played a decisive role in this process.

There are some limitations to the study. As direct measurements cannot be made to evaluate the nutritional status of infants, the amount of breast milk intake was calculated as an estimate based on the duration of breastfeeding. In order to determine breast milk consumption more accurately, the method of weighing infants before and after breastfeeding and basing their body weight on the change is recommended. However, this method could not be implemented because it is time‐consuming, and the time spent in the hospital is limited due to pandemic conditions. In addition, skin prick test applications planned to be carried out during the termination of the elimination diet could not be carried out due to pandemic restrictions. As in the diagnosis process, the oral food challenge test was used as the basis during the opening of the diet, and the process was carried out only with this method.

## 5 Conclusion

To minimize the negative effects of an elimination diet in children with cow's milk protein allergy, regular dietary assessments and nutritional counseling are essential. Monitoring growth, evaluating nutrient intake, and recommending supplements support healthy development. Individualized counseling aids breastfeeding and complementary feeding.

## Author Contributions

Conceptualization, M.N.Ç., E.K.; Data curation, M.N.Ç.; Formal analysis, M.N.Ç.; Project administration, M.N.Ç., E.K.; Software, M.N.Ç.; Supervision, E.K.; Writing – original draft, M.N.Ç.; Writing – review and editing, M.N.Ç., E.K. All authors have read and agreed to the published version of the manuscript.

## Disclosure

Institutional Review Board statement: The study was carried out according to the Declaration of Helsinki and it was approved by the Gazi University Ethics Commission with the date of 14.07.2020 and the meeting number 07.

## Consent

All parents who participated in the study signed an informed consent form before their infants were included in the study.

## Conflicts of Interest

The authors declare no conflicts of interest.

## Supporting information


Data S1

## Data Availability

The data that support the findings of this study are available on request from the corresponding author. The data are not publicly available due to privacy or ethical restrictions.
