# Supplementary material for: Individualized Nutritional Counseling Closes Growth and Nutrition Gaps in Infants With Food Protein‐Induced Allergic Proctocolitis: 6‐Month Follow‐Up Study
Source: Food Sci Nutr. 2025 Jun 20;13(6):e70498. doi: 10.1002/fsn3.70498 (PMC12181072; doi:10.1002/fsn3.70498)
Supplement: Supplementary file 1 — Data S1 [file FSN3-13-e70498-s001.docx]

**Supplementary Material**

S1. General plan of the study

|  | Baseline | Visit 1 (7th month) | Visit 2 (9th month) | Visit 3 (12th month) |
| --- | --- | --- | --- | --- |
| Healthy control/FPIAP | Questionnaire form  Anthropometric measurements  3-day food consumption record | | | |
| FPIAP | Training on transition to complementary feeding | Regulation of elimination diet | Oral food challenge  (Opening the diet) | Regulation of diet |
|  | Elimination of cow's milk from the mother's and infants’diet | Complementary feeding training per month | | |
| Healthy control | Training on transition to complementary feeding | Complementary feeding training per month | | |

FPIAP: Food Protein-Induced Allergic Proctocolitis

S2. Complementary Feeding Program

| **Age** | **Healthy controls** | | **FPIAP** | |  |
| --- | --- | --- | --- | --- | --- |
| 6 month | In addition to breastfeeding | Yogurt  Vegetable-fruit juices  Molasses  Egg yolk | | Vegetable-fruit juices  Molasses  Egg yolk | |
| 7 month |  | Whole egg yolk  Vegetable/lentil soup  Fruit/vegetable puree  Meat, chicken | | Whole egg yolk  Vegetable/lentil soup  Fruit/vegetable puree  Meat (Lamb meat can be preferred instead of beef due to cross-reactivity in babies diagnosed with CMPA. If lamb meat is also reactive, it is eliminated from the diet)  Chicken | |
| 8 month |  | Whole eggs  Pasteurized cheese  Fish  Grain and legume pastes | | Whole eggs  Fish  Cereal and legume pastes  Baked dairy products can be included as the diet is opened, depending on tolerance. | |
| 9-12 month |  | All meals cooked at home  Participation in the family table | | If the diet opening process is successful; all meals cooked at home  Participation in the family table  If the diet opening process is not successful, milk and dairy products are eliminated. | |

S3. Frequency, quantity and textural properties of complementary feeding

| Age (month) | Energy of complementary food (kcal/day) | Textural properties of complementary foods | Meal frequency (times/day) | Meal quantity (in grams)** | Breastfeeding frequency |
| --- | --- | --- | --- | --- | --- |
| 6-8 | 200 | Smooth-textured foods such as soups, thick purees, or well-mashed foods | 2–3 meals per day, with 1–2 additional snacks based on the child's appetite* | Start with 2–3 tablespoons per meal  150-200 mL | Continue breastfeeding on demand. |
| 9-11 | 300 | Semi-solid, lumpy foods, finely chopped items, and finger foods | 3-4 meals per day, with 1–2 additional snacks based on the child's appetite* | 210-280 mL |  |
| 12-23 | 500 | Family foods, chopped or mashed as needed | 3-4 meals per day, with 1–2 additional snacks based on the child's appetite* | 300-350 mL |  |

* Snacks are defined as foods that are eaten between meals, usually allowing for self-feeding, and are easy to prepare.

**The amount a baby can consume in a single meal depends on their gastric capacity, which can be estimated as approximately 30 mL per kilogram of body weight. Infants should not be expected to consume more than this amount at one time.

**Reference:** Sağlık Bakanlığı. (2016). Türkiye Beslenme Rehberi (TÜBER) [Turkey Nutrition Guide] (in Turkish). Sağlık Bakanlığı Yayınları, pp. 1–288.

World Health Organization. (2009). Infant and young child feeding: model chapter for textbooks for medical students and allied health professionals.
